# Analyzing Current Trends and Possible Strategies to Improve Sucrose Isomerases’ Thermostability

**DOI:** 10.3390/ijms241914513

**Published:** 2023-09-25

**Authors:** Amado Javier Sardiña-Peña, Liber Mesa-Ramos, Blanca Flor Iglesias-Figueroa, Lourdes Ballinas-Casarrubias, Tania Samanta Siqueiros-Cendón, Edward Alexander Espinoza-Sánchez, Norma Rosario Flores-Holguín, Sigifredo Arévalo-Gallegos, Quintín Rascón-Cruz

**Affiliations:** 1 Laboratorio de Biotecnología I, Facultad de Ciencias Químicas, Universidad Autónoma de Chihuahua, Circuito Universitarios s/n Nuevo Campus Universitario, Chihuahua 31125, Mexico; p324974@uach.mx (A.J.S.-P.); bfigueroa@uach.mx (B.F.I.-F.); mballinas@uach.mx (L.B.-C.); tsiqueiros@uach.mx (T.S.S.-C.); eaespinoza@uach.mx (E.A.E.-S.); sareval@uach.mx (S.A.-G.); 2 Laboratorio de Microbiología III, Facultad de Ciencias Químicas, Universidad Autónoma de Chihuahua, Circuito Universitarios s/n Nuevo Campus Universitario, Chihuahua 31125, Mexico; p324973@uach.mx; 3 Laboratorio Virtual NANOCOSMOS, Departamento de Medio Ambiente y Energía, Centro de Investigación en Materiales Avanzados, Chihuahua 31136, Mexico; norma.flores@cimav.edu.mx

**Keywords:** sucrose isomerases, protein structure, GH13 family, protein engineering, thermostability, thermostabilization

## Abstract

Due to their ability to produce isomaltulose, sucrose isomerases are enzymes that have caught the attention of researchers and entrepreneurs since the 1950s. However, their low activity and stability at temperatures above 40 °C have been a bottleneck for their industrial application. Specifically, the instability of these enzymes has been a challenge when it comes to their use for the synthesis and manufacturing of chemicals on a practical scale. This is because industrial processes often require biocatalysts that can withstand harsh reaction conditions, like high temperatures. Since the 1980s, there have been significant advancements in the thermal stabilization engineering of enzymes. Based on the literature from the past few decades and the latest achievements in protein engineering, this article systematically describes the strategies used to enhance the thermal stability of sucrose isomerases. Additionally, from a theoretical perspective, we discuss other potential mechanisms that could be used for this purpose.

## 1. Introduction

Enzymes are significant macromolecular biological catalysts, and much like conventional chemical catalysts, these biomolecules considerably accelerate chemical reactions by reducing their activation energy [1]. However, in contrast to chemical catalysts, enzymes offer appealing synthetic advantages, such as high selectivity, efficiency, sustainability [2,3,4], low catalyst loads [5], and the mild operating conditions required in some processes [1]. For these reasons, biocatalysis has emerged as a mature technology [6], transitioning from being of pure academic interest to a vital tool in the synthesis and manufacturing of chemicals [5,7,8].

Since the beginning of the 21st century, biocatalytic applications have seen an increase across many industrial sectors, ranging from fine and pharmaceutical chemistry to the manufacturing of chemical products and foods [9,10,11,12,13,14,15,16,17]. For instance, with a production of nearly 10 million tons of HFCS (High Fructose Corn Syrup) annually, immobilized glucose isomerase enzyme has been a commercial success [18]. Similarly, the enzymatic conversion of sucrose to isomaltulose using sucrose isomerases (EC 5.4.99.11) has emerged as a commercially attractive technology [19], currently reaching a production scale of more than 10,000 tons globally each year [20].

As isomaltulose (or Palatinose) stands as one of the potential candidates to replace sucrose as a food additive, its production has garnered significant attention worldwide [21,22]. Both sucrose and isomaltulose share similar physical and organoleptic properties; however, isomaltulose has about 50% of the relative sweetness of sucrose [23,24]. Compared to sucrose, isomaltulose is less labile under extreme pH conditions, demonstrating (in a 10% solution) stability for more than 30 min when incubated at pH 1.0 and 95 °C, while sucrose (in a 10% solution) nearly completely hydrolyzes under the same conditions [25]. In vivo studies indicate that isomaltulose is fully hydrolyzed and absorbed in the small intestine [26], meaning there are intestinal enzymes capable of fully hydrolyzing this carbohydrate. The hydrolysis rate, however, is much slower compared to sucrose and maltose [27,28,29]. Therefore, following the oral administration of isomaltulose, blood insulin, and glucose levels exhibit a slower rise and more prolonged and balanced residence times [27,30,31,32]. Moreover, isomaltulose intake is associated with enhanced fat oxidation compared to regular dietary carbohydrates. Thus, isomaltulose can increase the amount of fats utilized as an energy source, proving beneficial in preventing weight gain [26,32,33,34]. This suite of properties makes isomaltulose unique among the spectrum of alternative sweeteners currently marketed [25]. Nevertheless, isomaltulose occurs in extremely small amounts in nature, found only at about 1% in honey [35] and in sugarcane extracts [36]. Additionally, its synthesis through conventional chemistry is challenging [37]. Thus, sucrose isomerases (SIase), also known as isomaltulose synthase, sucrose α-glucosyltransferase, or trehalulose synthase, are pivotal for isomaltulose production [38].

Several microorganisms have been recognized for their SIase-producing capabilities. Among the microbial enzymes producing isomaltulose that have been purified and characterized are the isoforms from: *Erwinia* sp. [39,40], *Serratia plymuthica* sp. [41], *Protaminobacter rubrum* sp. [42], *Pantoea dispersa* sp. [43], *Klebsiella* sp. [44], *Enterobacter* sp. [45], *Agrobacterium radiobacter* sp. [46], *Pseudomonas mesoacidophila* sp. [47], and *Raoultella terrigena* sp. [48]. The genes encoding SIase have been designated as *palI* [39,41,49,50], *pal*-2 [48], *smuA* [42], *sim* [51], and *mutB* [52]. Specifically, the exponential development in genetic engineering has facilitated the recombinant expression of enzyme isoforms [39,42,49,50,51,52,53], with *Escherichia coli* being the most commonly employed host. Today, the availability of SIase expressed in food-grade strains is not a limitation in developing isomaltulose production processes. In fact, the low activity and stability of most SIase at temperatures above 40 °C is the bottleneck for their industrial application [54,55]. Precisely based on literature from recent decades and the latest advances in protein engineering, this article systematically outlines the strategies employed to increase the thermal stability of sucrose isomerases. Furthermore, from a theoretical standpoint, we discuss other mechanisms that might be employed for this purpose.

## 2. Sucrose Isomerases, Structure and Reaction Mechanism

**Figure 1:**
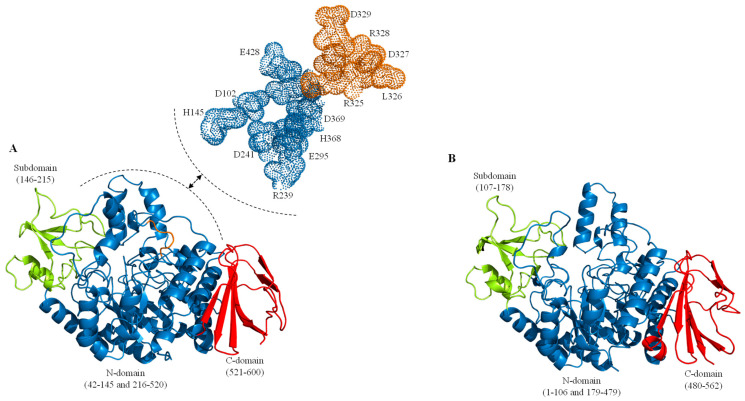
General structure of the GH13 family. (**A**) structure of the sucrose isomerase PalI NX-5. (**B**) structure of the trehalose-6-phosphate hydrolase. In (**A**,**B**), the molecules are displayed in the same orientation; the N-terminal catalytic (β/α)8 barrel is illustrated in blue, the subdomain in lime green, and the C-terminal domain in red. In (**A**), the isomerization region (residues 325–329) in PalI NX-5 is depicted in orange. The residues involved in the binding, hydrolysis, and isomerization of sucrose are shown enlarged.

The study of SIase began in the 1950s when the bacteria *Protaminobacter rubrum* CBS 574.77, which produces the SmuA isoform, was isolated and analyzed [42]. Today, in the Protein Data Bank (PDB, https://www.rcsb.org/, accessed on 13 June 2023), 28 crystal structures of SIase have been reported and resolved through X-ray diffraction (Table 1). Of these structures, 20 correspond to variants of the isoform from *Pseudomonas mesoacidophila* MX-45 [56,57,58], 5 to variants of the isoform from *Erwinia rhapontici* NX-5 [59], 2 to variants of the isoform from *P. rubrum* CBS574.77 [60], and 1 to the isoform from *Klebsiella* sp. LX3 [61]. These crystallographic data have elucidated that SIase are single-subunit molecules (Figure 1), with a central catalytic domain comprised of a (β/α)8 barrel similar to triose phosphate isomerases [56]. Furthermore, the architecture of the active site of SIase places them within the glycoside hydrolase family, family 13 (GH13) [61].

Established in the early 1990s, the GH13 family [62] represents the largest family of polysaccharide metabolizing enzymes [63,64], grouping enzymes with hydrolytic [65], transferase [66,67], and isomerase activities [68]. Specifically, differences in substrate specificity and/or enzymatic activities among the GH13 family members led to its subdivision into subfamilies. Today, according to the Carbohydrate-Active enZymes database (CAZy, http://www.cazy.org/, accessed on 13 June 2023), the GH13 family consists of 46 subfamilies [69]; however, this number continues to rise [70,71]. These subfamilies exhibit a clearer relationship between their sequences, enzymatic specificities, and phylogeny [72], which would facilitate predicting the catalytic function of enzymes with significant industrial potential from novel genes or microorganisms. Despite this apparent heterogeneity, GH13 enzymes are characterized by having three domains designated A, B, and C [61,73,74] (Figure 1). Specifically, for the NX-5 SIase, domain A (N-terminal catalytic) is located between residues 42–145 and 216–520 [59]. Likewise, the PalI isoform, the N-terminal catalytic domain, is located between residues 3–146 and 216–521 [61]. The N-terminal domain is a supersecondary (β/α) structure of eight barrels, which is the main body of the SIase (the enzyme’s catalytic center). The subdomain (domain B) between residues 146–215 (positions in the NX-5 and PalI isoforms) is a structure rich in short loops, with no known function in PalI or other GH13 family members. Lastly, the C-terminal domain located between residues 521–600 (position in the NX-5 isoform) consists of two antiparallel β-sheets; this domain interacts with the N-terminal domain through the formation of salt bridges and hydrogen bonds. Thus, domain C is associated with the structural stability of SIase [59,61].

**Figure 2:**
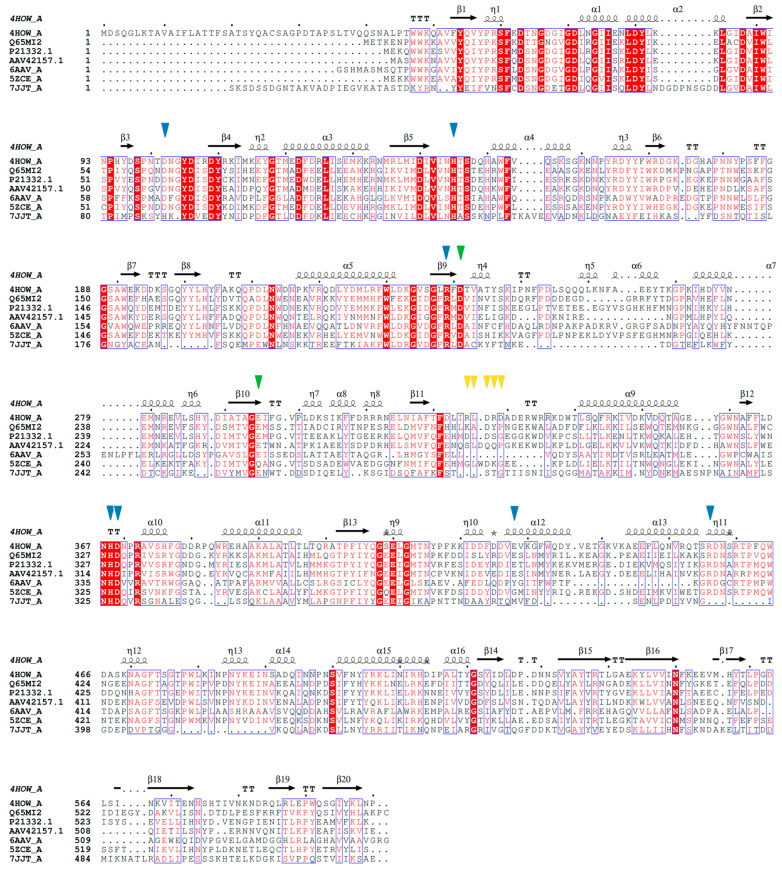
Multiple sequence alignment of the sucrose isomerase PalI NX-5 (Swiss-Prot: 4HOW_A) and other enzymes from the GH13 family. Amino acid sequences of the trehalose-6-phosphate hydrolase from *Bacillus licheniformis* (Swiss-Prot: Q65MI2), the oligo-1,6-glucosidase from *Bacillus cereus* ATCC7064 (Swiss-Prot: P21332.1), the 1,6-α-glucosidase from *Lactobacillus acidophilus* NCFM (Swiss-Prot: AAV42157.1), the α-glucosyl transfer enzyme XgtA from *Xanthomonas campestris* WU-9701 (Swiss-Prot: 6AAV_A), the α-glucosidase BspAG13_31A from *Bacillus* sp. AHU2216 (Swiss-Prot: 5ZCE_A), and the α-amylase from *Ruminococcus bromii* (Swiss-Prot: 7JJT_A) are displayed. The residues from PalI NX-5 involved in sucrose binding are indicated with a blue triangle, residues crucial for sucrose hydrolysis are highlighted with a green triangle, while residues involved in isomerization and specificity towards isomaltulose formation are denoted with an orange triangle. Sequence alignments were generated using the software MEGA 11 Version 11.0.13 [83] and ESPript [84]. Secondary structures are designated according to the crystal structure of PalI NX-5 and are presented on top: helices with squiggles, beta strands with arrows, turns with TTT letters. Residues depicted or highlighted in red within the blue blocks represent conserved residues.

Among the GH13 enzymes, those most structurally similar to SIase include: the oligo-1,6-glucosidase from *Bacillus cereus* ATCC7064 [75], the 1,6-α-glucosidase from *Lactobacillus acidophilus* NCFM [76], the trehalose-6-phosphate hydrolase from *Bacillus licheniformis* [77], the α-glucosyl transfer enzyme XgtA from *Xanthomonas campestris* WU-9701 [78], the α-glucosidase BspAG13_31A from *Bacillus* sp. AHU2216 [79], and even the α-amylase from *Ruminococcus bromii* [80] (Figure 1 and Figure 2). This structural similarity suggests a similar substrate binding and catalysis mechanism among GH13 enzymes. Specifically, GH13 enzymes share the same double displacement catalytic mechanism, proceeding via the accumulation and subsequent decomposition of a glycosyl-enzyme intermediate [81]. Additionally, the catalytic machinery of the GH13 family comprises a triad of residues: a catalytic nucleophile (aspartic acid), a proton donor (glutamic acid), and a transition state stabilizer (aspartic acid) [82].

**Figure 3:**
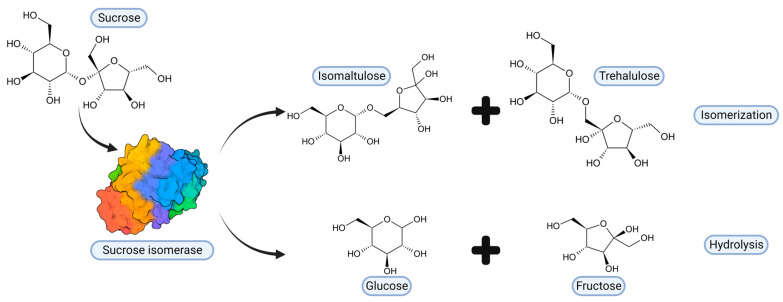
Hydrolysis and isomerization of sucrose catalyzed by SIase.

Among SIase, five highly conserved residues present in the catalytic N-terminal domain stand out. Zhang et al. [61] point to Asp241, Glu295, and Asp369 (position in the PalI isoform) as the potential catalytic triad, while His145 and His368 are highly conserved in α-amylases and glycosyltransferases [85]. In PalI SIase, Glu295 acts as a general acid by protonating the oxygen of the glycosidic bond and producing substrate hydrolysis; then, the Oδ2 of Asp241 acts as a nucleophile and attacks the C1 of D-glycosyl, forming the β-glycosyl-enzyme intermediate, while Asp369 forms hydrogen bonds with O2 and O3. His145 forms a hydrogen bond with O6 and His368 with O2. Isomaltulose is produced when the O6 of D-fructose (hydrolysis product) nucleophilically binds to the C1 of the D-glycosyl group [61]. Unlike the GH13 enzyme family, among SIase, the RLDRD motif (RYDRA, in the MutB isoform) is highly conserved. The sequence 325RLDRD329 (position in the PalI isoform) is adjacent to the active site cleft. Through directed mutagenesis analysis of this sequence, its indispensable and determinant role in enzymatic kinetics and specificity towards isomaltulose formation has been confirmed [61,86]. Based on modeling studies, it is suggested that the RLDRD sequence, especially the two arginines, is involved in binding to fructose [86]. In this way, SIase possesses both isomerase and hydrolase activities, although the amounts of D-glucose and D-fructose are minimal [43]. Thus, isomaltulose and trehalulose are the primary products of the enzyme’s catalytic action [29] (Figure 3).

**Figure 4:**
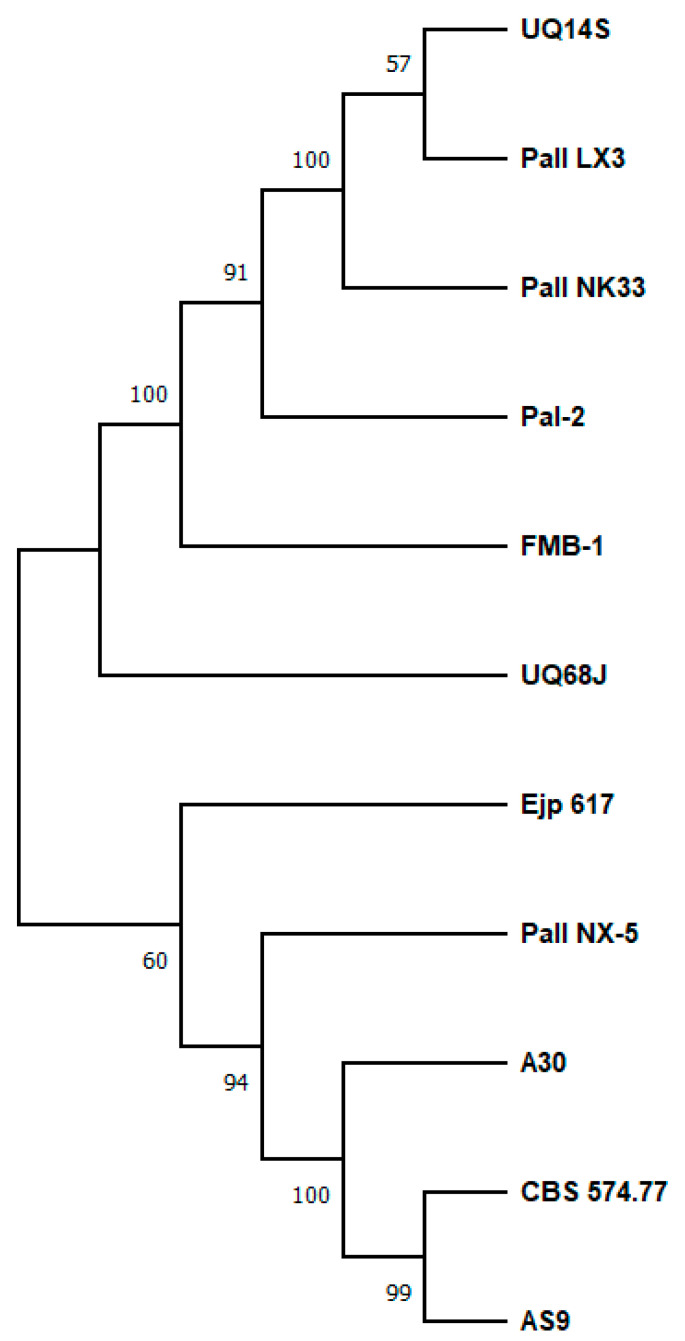
Phylogenetic tree of sucrose isomerase isoforms. A consensus phylogenetic tree was constructed using the the software MEGA 11 Version 11.0.13 [83]. The evolutionary history was inferred using the Maximum Likelihood method and the JTT matrix-based model. The tree with the highest log likelihood (−5484.74) is displayed. The percentage of trees in which the associated taxa clustered together is indicated next to the branches. Initial tree(s) for the heuristic search were automatically obtained by applying the Neighbor-Join and BioNJ algorithms to a matrix of pairwise distances, which were estimated using the JTT model. Then, the topology with the superior log likelihood value was selected. This analysis included 11 amino acid sequences. The final dataset comprised 600 positions. The sequences used belong to the isoforms: PalI NX-5 (GenBank: ADJ56407), CBS 574.77 (GenBank: 3GBE_A), PalI NK33 (GenBank: AAM96902.1), UQ68J (GenBank: AAP57083.1), UQ14S (GenBank: AAP57085.1), PalI LX3 (GenBank: 1M53_A), A30 (GenBank: EKF64560.1), AS9 (GenBank: ALS09706.1), Ejp617 (GenBank: ADP12651.1), FMB-1 (GenBank: ACF42098.1), and Pal-2 (GenBank: VUC84579.1).

Despite the high homology and structural similarity among SIase, their catalytic behavior varies considerably depending on the analyzed isoform, pH, and reaction temperature (Figure 4, Table 2). For instance, the isoform from *P. dispersa* UQ68J produces up to 91% isomaltulose and 3% trehalulose (30–35 °C) [43], while the SIase from *A. radiobacter* MX-232 [46] and *P. mesoacidophila* MX-45 [47] yield up to 88% trehalulose. Significant differences also exist in the Michaelis–Menten constants (Km) and catalytic efficiencies of various SIase (Table 2). Thus, Km is estimated in the range of 30.1 mM (recombinant isoform from *Serratia plymuthica* AS9) [55] to 255.1 mM (recombinant isoform from *E. rhapontici* NX-5) [54]. Meanwhile, catalytic efficiencies (k_cat_/Km) have been reported in the range of 1301 mM^−1^·s^−1^ (recombinant isoform from *P. rubrum* CBS 574.77) [86] to 0.27 mM^−1^·s^−1^ (recombinant isoform from *Klebsiella* sp. LX3) [85].

## 3. Thermolability of Sucrose Isomerases

With the exception of the isoform from *Enterobacter* sp. FMB-1 (T_op_ 50 °C) [50], most SIase exhibit their peak activity between 20 and 40 °C [89,90] (Table 2). Even so, the isoform from *Enterobacter* sp. FMB-1 dramatically loses its activity at temperatures exceeding 50 °C [50]. Similarly, the recombinant isoform from *E. rhapontici* NX-5 displayed no activity after being incubated for 30 min at 60 °C [87], while the wild-type variant has a half-life of 5 min at the same temperature [39]. For its part, the isoform from *Erwinia* sp. Ejp617 retained only 1.7% of its peak activity after 1 h of incubation at 50 °C [89]. Likewise, the recombinant isoform from *Pantoea dispersa* lost up to 71% of its activity after 1 h of incubation at 45 °C [91]. Thus, the high thermolability of SIase presents a challenge in the industrial production of isomaltulose [55].

The issue of enzyme instability has posed a fundamental challenge in their use for productive-scale synthesis and chemical production [92]. Often, harsh reaction conditions are required, such as elevated temperatures and exposure to organic solvents [3]. Reaction rates increase exponentially with temperature, reaching a point of enzyme denaturation [93]. Thus, their thermal stability becomes a desirable attribute in the development of an industrial process. Additionally, enhanced thermostability is also associated with longer half-lives under mild conditions and greater retention of activity in non-aqueous solvents [94]. These properties, combined with a suitable reaction strategy, would facilitate the reuse of the biocatalyst and thereby reduce the costs associated with the operation of the process [95,96,97]. This approach also addresses the cost stigma associated with enzyme use [98]. Specifically, the utilization of thermally stable SIase enzymes would offer benefits; as temperature increases, there is a decrease in the viscosity of the reaction mixture, enhanced substrate solubility, increased mass transfer rates, and a reduction in the risk of microbial contamination [55,99,100].

Several structural elements contribute to the thermal stability of enzymes. Numerous studies addressing the crystal structures of mesophilic and thermophilic proteins have demonstrated the pivotal relationship between structure and thermostability [92], further identifying key factors affecting enzyme thermostability [101]. Among these, the composition of amino acids forming their helices stands out [102,103]. For instance, when comparing the prevalence of Tyr, Gly, and Gln in helices of thermally stable proteins, these amino acids appear in greater abundance than in mesophilic proteins, whereas the opposite is true for Val [104]. Substitutions such as Lys → Arg and Ser → Ala have also been observed to be common when comparing mesophilic to thermophilic proteins [105]. Other core determinants of protein thermal stability include increased hydrogen bonding [106,107,108], the introduction of disulfide bonds [109,110,111], salt bridges [112,113], loop shortening [114,115], optimization of electrostatic surfaces [116,117], enhanced solvation in specific protein regions [118], and augmented intramolecular hydrophobic packing [119,120]. The factors mentioned previously largely share a similar basis, aiming to reduce the molecule’s entropy; that is, enzyme rigidity is required for higher thermostability [108]. It should be noted that the enhancement of thermal stability is achieved through the cooperative optimization of various factors rather than a single dominant interaction [121].

## 4. Protein Engineering, Thermostabilization of Sucrose Isomerases

**Figure 5:**
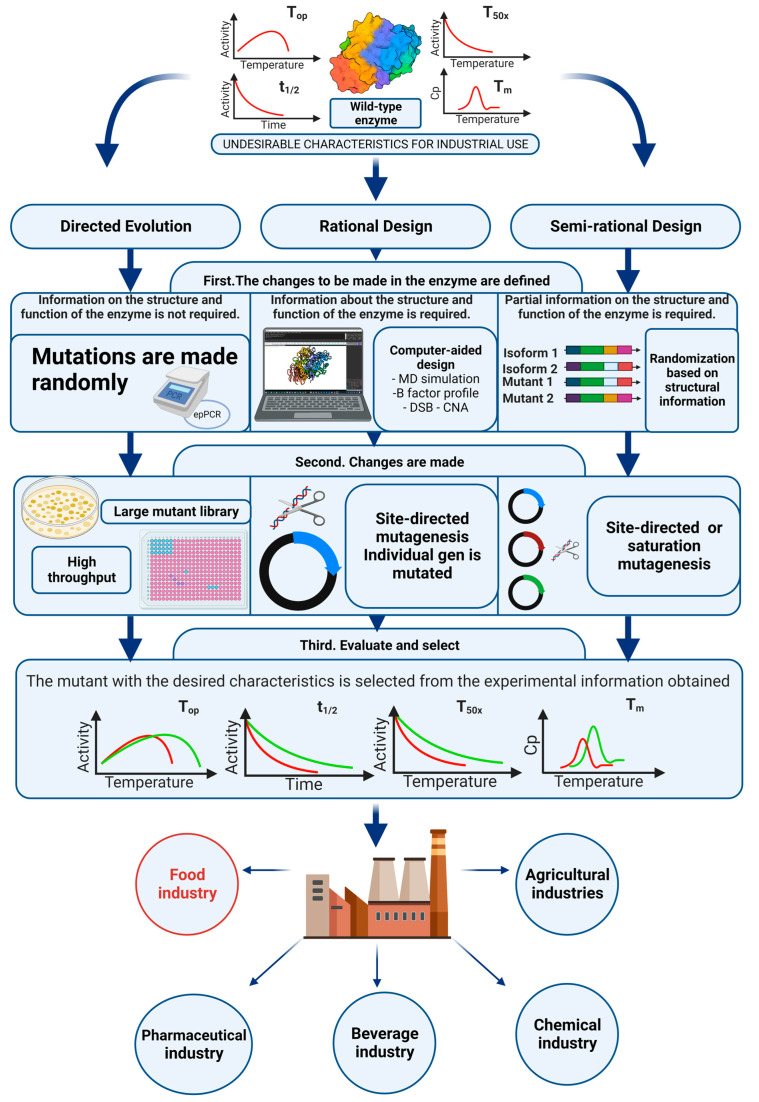
Protein engineering strategies in the thermostabilization of enzymes.

Protein engineering is a widely used method in the stabilization of mesophilic enzymes [92,122]. In this sense, the use of protein engineering tools accumulates several industrial successes, some of which date back to the 1980s when Genencor designed bleach-tolerant proteases for laundry detergents [123]. Numerous strategies have been developed to enhance protein thermostability, which typically falls into three approaches: rational design; directed evolution; and semi-rational design [55,124,125,126]. The choice of strategy will depend on both the availability of a robust screening method and information about the enzyme’s structure and function [127]. Broadly speaking, engineering to enhance an enzyme’s properties follows three steps: initially, changes to be made in the protein are defined using one of the engineering strategies; subsequently, the proposed changes (mutagenesis) are implemented; and finally, protein variants are evaluated to select mutants with superior properties [128] (Figure 5).

### 4.1. Directed Evolution

Directed evolution, proposed by Arnold in the 1990s, mimics the process of natural evolution. This strategy encompasses genetic diversification, screening, and the selection of valuable mutants from a vast array of mutant libraries [129,130]. The error-prone PCR (epPCR) technique, vital within the context of directed evolution, is a highly effective method for generating mutants with enhanced thermostability [92]. In this method, during the replication process, a modified DNA polymerase introduces random mutations into the gene of interest [131,132]. Hence, practicing directed evolution takes on a quid pro quo nature, as its execution does not necessitate prior knowledge of the target enzyme’s structure and function or the various amino acid substitutions generated [133]. This strategy’s primary challenge lies in the vast number of mutant colonies produced (typically around 10^4^) [124]. A robust screening and selection method for mutants with desired characteristics is required, rendering the process time-consuming and costly [134]. There are also limitations in constructing highly diverse mutant libraries, such as the commonly low mutagenic frequency, the redundancy of the genetic code, and mutagenic “hot spots” caused by the propensity of polymerases [135].

Rational design mandatorily requires detailed structural information that might not yet be available for all enzymes [92]. In this way, directed evolution could be applied to proteins lacking structural research, and the effectiveness of this strategy has ensured its popularity, even as computer-assisted engineering plays a significant role in enzyme thermostabilization. Pertinently, enzymatic engineering through directed evolution, although not applied to sucrose isomerases, has successfully enhanced the thermostability of GH13 enzymes such as the α-glucosidase from *Thermus thermophilus* TC11 [136] and the α-amylase from *Bacillus licheniformis* [137].

### 4.2. Rational Design

Rational design is rooted in understanding the relationship between a protein’s structure and function [138]. Based on this relationship, precise changes are introduced into the amino acid sequence through site-directed mutagenesis [133]. In this regard, rational design and understanding of the structure–function relationship have evolved synergistically, thus opening new perspectives for modulating enzymatic function and de novo prediction [139]. These attributes give rational design the potential to drastically reduce the size of the mutant library and the associated screening costs [92]. In recent years, with the development of bioinformatics, numerous algorithms and computational tools have emerged that allow the monitoring of flexible regions in protein molecules, as well as predicting thermostabilization [140,141]. Precisely, computer-assisted rational design is an attractive alternative that speeds up the process of enzymatic engineering [142]. Commonly, in the in silico stabilization of enzymes, strategies are followed such as comparison with homologous sequences of greater thermostability [143], analysis of the B-factor [144,145], molecular dynamics (MD) simulations [113,146], constraint network analysis (CNA) [147], designing disulfide bonds (DSB) [142,148,149], engineering glycosylation sites [150], designing stabilizing salt bridges based on enzyme sequence and structure [151], and calculations to minimize effective energy that mimics Gibbs free energy [152,153]. During the application of these strategies, substitutions made should not belong to a stabilization center or the active site cavity. Thus, mutations are often observed in loop regions or exposed surface areas [110,115].

### 4.3. Semi-Rational Design

Using the primary structure of a protein to predict biochemical and biophysical parameters is an attractive field of research. This is because information on genomic sequencing is expanding much more rapidly than structural or biochemical data [154]. For this reason, semi-rational design has emerged as a highly attractive strategy in which directed evolution and rational design are combined. This combination allows for the reduction of mutant library sizes [133]. Additionally, considering evolutionary variability, mechanical features, and topological limitations for amino acid identification can result in libraries having a higher functional content [155].

### 4.4. Characterization of Thermostability

Frequently, the thermostability of an enzyme is characterized by quantifying the physical parameters: optimal temperature (T_op_) and its associated activity range [120], melting point temperature (T_m_) [119], the T_50x_, and the half-life time (t_1/2_) [156]. T_m_ characterizes the irreversible unfolding of the protein’s secondary or tertiary structure, being one of the most informative parameters of thermostability [157]. On the other hand, T_50x_ represents the temperature at which half of the residual activity remains after a time “x” (min); this parameter is an indicator of temperature-dependent deactivation. The t_1/2_ parameter represents the time the enzyme retains half of its residual activity at a specified temperature. Thus, it is a kinetic parameter of stability [120]. Figure 5 provides a summarized depiction of the aforementioned aspects.

### 4.5. Thermostabilization of Sucrose Isomerases

Protein engineering techniques applied to SIase have targeted three primary goals: enhancing enzyme thermostability, boosting enzyme activity, and increasing the isomaltulose production ratio (Table 3). Zhang et al. [85] honed in on improving thermostability by identifying potential sites for proline substitution. It is recognized that the presence of proline in the second position of a β-turn makes a protein more stable by reducing its entropy [158,159]; this concept has been extensively applied for enhancing thermostability in α-amylases [159,160]. Zhang et al. [85] chose the residues Arg310 and Glu498 for proline substitution. Resulting from the mutations, the optimal temperature of PalI increased from 35 °C to 40 °C and 45 °C for PalI:Glu^498^Pro and PalI:Glu^498^Pro/Arg^310^Pro, respectively. The half-lives of PalI, PalI:Glu^498^Pro, and PalI:Glu^498^Pro/Arg^310^Pro were 1.81, 9.45, and 13.61 min at 50 °C, respectively. Thus, the half-life of PalI:Glu^498^Pro/Arg^310^Pro at 50 °C was approximately 11 times higher than that of PalI. Similarly, to enhance the stability of the *S. plymuthica* AS9 isoform, Duan et al. [55] identified amino acid residues with high B-factors for site-directed mutagenesis. The mutants E^175^N, K^576^D, and E^175^N/K^576^D were designed using the Rosetta Design database. As a thermostability enhancement result, the mutants displayed a slightly increased optimal temperature (35 °C) compared to the wild-type enzyme (30 °C). The half-lives of mutants E^175^N, K^576^D, and E^175^N/K^576^D were 2.30, 1.78, and 7.65 times longer than the wild-type enzyme at 45 °C, respectively. Meanwhile, Sardiña-Peña et al. [54] enhanced the thermal stability of the *Erwinia rhapontici* NX-5 isoform. The authors aimed to enhance the presence of thermostabilizing interactions, such as hydrogen bonds and glycosylation, in the molecule’s flexible regions. The engineered mutants K^174^Q, L^202^E, and K^174^Q/L^202^E were expressed as glycoproteins and exhibited an increase in their optimal temperature by 5 °C, while their half-lives at 40 °C increased by factors of 2.21, 1.73, and 2.89, respectively.

When collectively analyzing the preceding reports, the following similarities stand out. Firstly, mutation sites (substituted residues) were located on the molecules’ periphery (surface), which is undoubtedly associated with this region being the most susceptible to changes in the enzyme’s microenvironment. Secondly, target sites corresponded to loop regions with significant flexibility. Duan et al. [55] and Sardiña-Peña et al. [54] laid the foundation for their strategies by interpreting the B-factor profile of the crystal structures 3GBD (Resolution 1.95 Å) and 4HOW (Resolution 1.7 Å, Table 1), respectively. These profiles are a practical and helpful tool for identifying flexible regions (hotspots) in any enzyme. However, regarding B-factors as a sufficient foundation for flexibility conclusions could pose risks and errors [161]. Merritt [162] argues that the crystal structure’s resolution is a pivotal parameter when using the B-factor. A low resolution (3–5 Å) correlates with disproportionate B-factors, which should not be used for specific conclusions. Even if the resolutions of the SIase crystal structures are relatively high, drawing factual conclusions from the B-factor might remain limited [163].

The strategies delineated by Zhang et al. [85], Duan et al. [55], and Sardiña-Peña et al. [54] were not aimed at enhancing the enzymes from a kinetic standpoint. However (Table 3), as reported by Zhang et al. [85], compared to PalI, the maximum specific activity increased by 7% for PalI:Glu^498^Pro and by 16% for PalI:Glu^498^Pro/Arg^310^Pro. Duan et al. [55] report that in comparison to PalI AS9, the Km values for the mutants E^175^N, K^576^D, and E^175^N/K^576^D decreased by 6.6%, 2.0%, and 11.0%, respectively, and their catalytic efficiency values increased by 38.2%, 4.2%, and 19.4%, respectively. Similarly, as reported by Sardiña-Peña et al. [54], the Km values for the mutants K^174^Q, L^202^E, and K^174^Q/L^202^E decreased by 5.1%, 7.9%, and 9.4%, respectively; additionally, the catalytic efficiency increased by up to 16%. Furthermore, the mutants exhibited an increase in activity from 20.3% to 25.3%. Both Duan et al. [55] and Sardiña-Peña et al. [54] justified the increased activity by stating that the substitutions made were distanced from the catalytic center and isomerization region. Therefore, the mutations might have caused these regions to be more compact, exerting a positive impact on kinetic parameters. In light of this, Liu et al. [48] enhanced the enzymatic activity of the *Raoultella terrigena* isoform using a thermal stability optimization strategy. For this purpose, they employed the Hotspot Wizard tool (https://loschmidt.chemi.muni.cz/hotspotwizard/, accessed on 15 May 2021) [164], which enabled the identification of hotspots through multiple sequence alignment. Compared to the wild-type PalI-2, the enzymatic activities of the mutants N^498^P and Q^275^R increased by 89.2% and 42.2%, respectively, while the isomaltulose production efficiencies of the mutants Y^246^L, H^287^R, and H^481^P improved up to 89.1%, 90.7%, and 92.4%, respectively.

## 5. Glycosylation of Sucrose Isomerases

**Figure 6:**
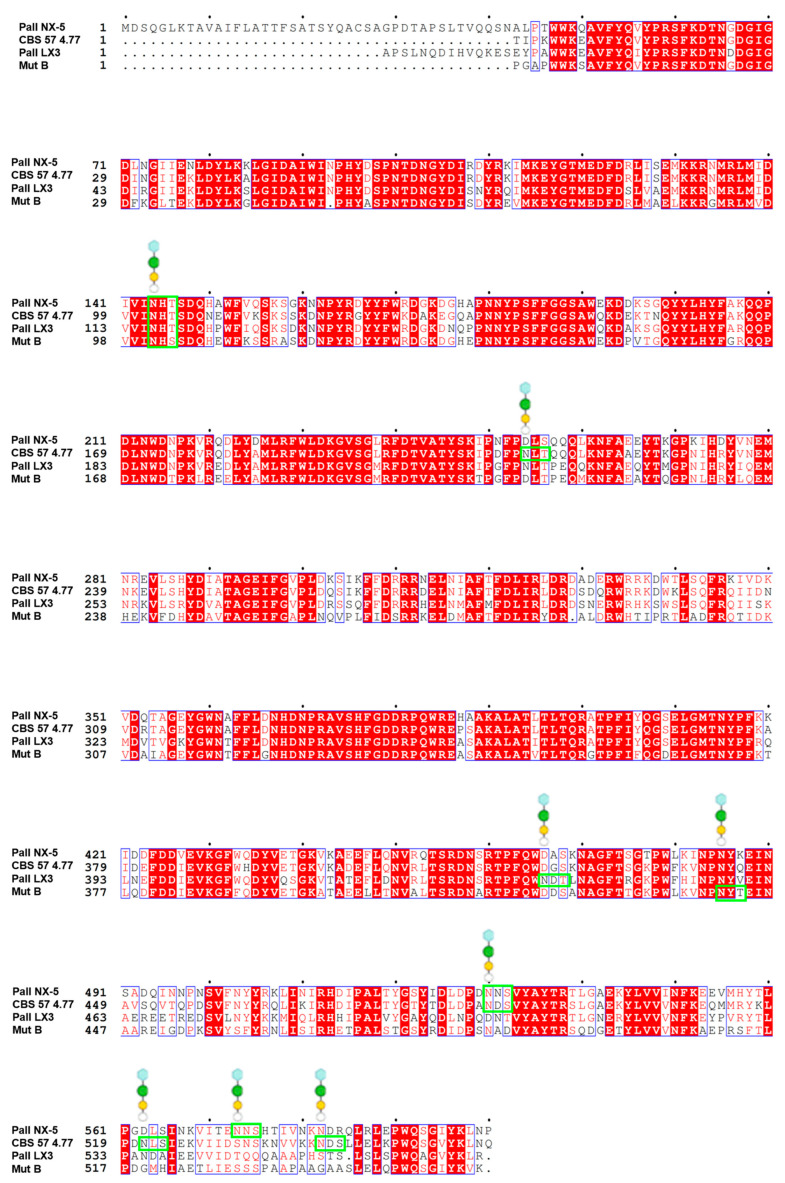
Indicated in green boxes are the potential sites for N-glycosylation present in the PalI NX-5, CBS 574.77, PalI LX3, and MutB isoforms. Monosaccharides point to the glycosylable asparagine residue, monosaccharide symbols follow the SNFG system. Residues depicted or highlighted in red within the blue blocks represent conserved residues. Sequence alignments were generated using the software MEGA 11 Version 11.0.13 [83] and ESPript [84].

SIase are molecules naturally produced in prokaryotes. Due to this, the potential effects of post-translational modifications, such as glycosylation, on the structure, function, and stability of SIase have not been deeply studied. However, if a protein naturally produced in prokaryotes possesses potential sites for N-glycosylation, its expression in a eukaryotic host could result in a glycoprotein. As shown in Figure 6, SIase isoforms such as PalI NX-5, CBS 574.77, PalI LX3, and MutB display potential sites for N-glycosylation. Specifically, Sardiña-Peña et al. [54] took this into account during the thermostabilization of the PalI NX-5 isoform by choosing *Pichia pastoris* as the expression system. Both the non-transformed variant and the mutants developed by Sardiña-Peña et al. [54] were expressed as glycoproteins. Nevertheless, Sardiña-Peña et al. [54] did not deeply analyze the effect that glycosylation might have had on the molecule’s thermostability and kinetic behavior. However, it can be noted that in the case of the PalI NX-5 mutant variants (K174Q, L202E, and K174Q/L202E), the observed increase in thermostability could have resulted from the simple effect of the introduced mutations or their combination with the molecule’s glycosylation. While the non-mutated but glycosylated variant showed the same T_op_ as reported by Ren et al. [87] (Table 2 and Table 3), the apparent effect of glycosylation on the T_op_ was null. Yet, from a kinetic perspective, the glycosylated PalI NX-5 displayed enhanced enzymatic activity.

N-glycosylation is one of the most common co- and post-translational modifications in eukaryotes [165]. This modification usually occurs in asparagine residues within the consensus sequence Asn-X-Ser/Thr, where X cannot be proline because the pyrrol ring structure of proline increases the rigidity of the peptide chain and inhibits glycosylation [166]. In this sense, Knauer and Lehle [167] estimate that between 70–90% of the consensus sequences present in secreted proteins have glycans. However, when the folding of the polypeptide chain occurs in the cytosol, not necessarily all consensus sequences are accessible to the action of glycosyltransferases to start glycosylation [168], which would happen among the SIase. As seen in Figure 1A, Figure 2 and Figure 6, potentially glycosylate regions like the one located in the enzyme’s catalytic cavity (N144 in PalI NX5, N102 in CBS574.77, N116 in PalI LX3, and N102 in MutB) would be discarded if the action of glycosyltransferases occurred after enzyme folding. It should be noted that N-glycosylation begins when the oligosaccharide precursor (Glc3Man9GlcNAc2) is transferred, through oligosaccharide transferase, to the consensus sequence present in the nascent polypeptide [169]. The N-glycans of proteins are subsequently processed by a series of glucosidases and glycosyltransferases in the endoplasmic reticulum and the Golgi apparatus [170].

Protein glycosylation typically constitutes a stabilizing interaction against temperature, pH, the presence of proteases, and physiological stress [171,172,173,174]. In this regard, Hu et al. [175] suggest that glycosylation reduces the flexibility of the protein structure, thereby enhancing its structural and thermal stability. Furthermore, studies by Ryu et al. [168] and Maksimainen et al. [176] indicate extensive interactions of immature N-glycans with their carrier proteins. Moreover, glucose residues on the non-reducing end exhibit extensive interactions with the protein surface [177]. Interactions have even been observed between glycans and residues located 30 Å from the N-glycosylated Asn [178]. Thus, N-glycosylation might affect the protein’s stability [150,179], activity [144], and specificity [180].

The effect of glycosylation is complex and not always predictable or beneficial [150,175]. Such behavior has been noted within the GH13 family. For instance, Hu et al. [175] observed that the glycosylation of α-amylase BLA from *Bacillus licheniformis* had no significant impact on the molecule’s thermal stability since the inherent stability of BLA overshadowed the glycosylation effect on its thermal stability. Assessing the effect of glycosylation on enzyme thermal stability becomes even more complex when considering the various glycosylation patterns obtainable in eukaryotic hosts. In yeasts, protein N-glycans undergo processing to form structures rich in mannose. However, the fully processed forms of the N-glycan vary depending on the species [181]. Moreover, for a given microorganism, the glycosylation process might be influenced by environmental factors such as fermentation duration, the expression level of glucosidases/glycosyltransferases, and culture medium composition [182]. Additionally, intrinsic structural factors associated with the nascent protein also affect glycan processing, maturation, and subsequent interaction with the side chain [183]. Given the aforementioned discussions, analyzing the effect of N-glycosylation on sucrose isomerases is intriguing, especially considering that they naturally possess multiple consensus sequences (Figure 6).

## 6. Future Perspectives

Even though the thermostability of SIase is a limiting factor and multiple reports have deeply examined their structure–function relationship [56,59,60,61,86,184], research focused on enhancing the thermal stability of SIase using protein engineering is quite limited and leaves questions regarding potential strategies that could be employed in this direction. Therefore, the following discussion, from a theoretical perspective, considers other mechanisms that might be used for this purpose.

### 6.1. Thermostable Sucrose Isomerases Based on Homology Models and Chimerization

In contrast to SIase, some members of the GH13 family exhibit elevated optimal temperatures, often exceeding 70 °C. Hence, these enzymes have been widely used in the formulation of detergents, the baking industry, beer production, alcohol manufacture, and starch sugar production [175]. For example, the thermostable α-amylase from *Bacillus licheniformis* B4-423 displays an optimal temperature of 100 °C. This enzyme can maintain more than 70% activity in the 80–140 °C range. Likewise, this α-amylase retained over 50% of its enzymatic activity after incubation at 80 °C for 110 min [185]. Also noteworthy are the α-amylases from *Pyrococcus furiosus* [186] and *Bacillus amyloliquifaciens* TSWK1-1 [187], with optimal temperatures of 100 °C and 70 °C, respectively. In both cases, they exhibited half-lives of 12 h at their optimal temperatures. The 1,4-α-glucan branching enzyme from *Bacillus licheniformis* ATCC14580 displays peak activity at 80 °C and retains 90% of its enzymatic activity at 70 °C over 16 h. Similarly, the trehalose synthase from *Thermobaculum terrenum* ATCCBAA-798 presents an optimal temperature of 45 °C, also retaining 80% of its peak activity after treatment at 70 °C for 30 min [188].

The characteristics of the thermostable GH13 enzymes make them an unexplored niche for designing SIase with novel properties. Consider that the GH13 family displays a highly conserved structure and mechanism among its members. Moreover, the structure–function relationship of SIase has been thoroughly examined. In this context, both homology models (sequence-based) and structure-based designs could serve as alternatives for detecting “hotspots” affecting thermal stability. Such rational design approaches, followed by in silico structural analysis, would facilitate the creation of mutant and chimeric SIase with enhanced hydrogen bonding and hydrophobic interactions. For instance, Cui et al. [143] employed a sequence-based design to enhance the specific activity and thermal stability of the α-amylase BLA from *Bacillus licheniformis*. Using this approach, Cui et al. [143] designed the Q^360^C mutant, which exhibited a residual activity 1.27 times higher than the wild-type variant after pre-incubation at 70 °C for 30 min. This result suggests that an approach grounded in multiple sequence alignment is feasible for the thermo-stabilization of other GH13 family enzymes, like SIase.

### 6.2. Thermostable Sucrose Isomerases Based on Improving the Entropy of the Folded State

It has been observed that modifications enhancing the entropy of the folded state can also lead to stabilization [189]. Specifically, strategies such as truncation or cyclization of mesophilic enzymes have employed this paradigm. In this context, it has been reported that the removal of exposed loop regions can enhance stability. Studies on flexible loop areas have shown that the entropic effect of ordering a loop upon folding is analogous to the energetic outcomes obtained after loop truncation [190]. Furthermore, when comparing mesophilic or thermophilic structures, an inverse correlation has been observed between loop length and stability, and additionally, increased stability through loop shortening [191]. Although truncation has not yet been implemented for SIase thermostabilization, this approach has been pursued with some members of the GH13 family, such as the alkaline α-amylase (Amy703) from *Bacillus pseudofirmus* 703. This enzyme was truncated in the N-terminal domain by Lu et al. [192], who developed the N-Amy mutant. This mutant displayed a T_op_ of 50 °C, 10 °C higher than Amy703. Thus, the potential thermostabilization of SIase by truncating segments of domain B (loop-rich) becomes highly intriguing for future work. Beyond this, conducting protein engineering activities on domain B might assist in discerning its function within the SIase. In this regard, studies like those by Feller et al. [193] and Rhimi et al. [194] have employed truncation of segments with flexible loops in GH13 enzymes to elucidate their role in inhibitor tolerance and enhancement of enzymatic activity.

Cyclization is another strategy to enhance the entropy of a protein’s folded state. Broadly speaking, cyclization aims to connect the N and C termini of a protein’s amino acid chain. Typically, the N and C-terminal regions are often the most flexible parts of the protein’s backbone [195]. Protein backbone cyclization can be achieved through chemical ligation, the introduction of disulfide bonds, and peptide ligation [156]. Particularly, protein cyclization using biological conjugation approaches like the SpyTag/SpyCatcher and SnoopTag/SnoopCatcher systems has been a highly effective strategy for creating thermostable enzymes [196]. The Tag/Catcher system leads to specific covalent conjugation of the protein backbone through two short polypeptide tags via the formation of an isopeptide bond between two amino acid side chains [197]. In the context of the GH13 family, enzymatic cyclization has been successfully developed, as seen in the research conducted by Chen et al. [146] on the thermostability of trehalose synthase (TreS) from *Thermomonospora curvata*. In their study, Chen et al. [146] noted that in the case of TreS, cyclization led to a much greater increase in thermostability than was achieved through site-directed mutagenesis. It is worth noting that, to date, approaches to SIase thermostabilization have primarily focused on site-directed mutagenesis. Thus, it would be interesting to evaluate the effect of a cyclization-based approach. However, it should be emphasized that in protein engineering, there are no universal strategies. The introduction of inteins can destabilize a protein due to steric constraints in the folded conformation, limiting the broad application of protein cyclization [157].

Whether within the framework of cyclization or as an independent strategy, disulfide bond engineering is also a successful approach for enzyme stabilization [149]. However, the reasons behind the stabilizing effect of this bond are not well characterized, and contrary to expectations, many designed disulfide bonds have resulted in reduced stability of the modified protein [198]. In some cases of GH13 enzymes, such as SIase, the C-terminal domain is associated with structural stability [59,61]. Precisely with this in mind, Li et al. [199] introduced a disulfide bridge in the C-terminal domain of the α-amylase (FSA) from *Flavobacteriaceae sinomicrobium*. They achieved this by making the S^450^C and K^415^C substitutions, leading to a significant improvement in enzyme activity and thermostability. Thus, introducing disulfide bridges in the C-terminal domain, or even in the loop-rich domain, might be a feasible strategy in the thermostabilization of SIase.

### 6.3. Thermostable Sucrose Isomerases, Other Alternative Strategies

Beyond protein engineering, strategies based on chemical modifications also allow for the enhancement of enzyme stability. For instance, PEGylation has been used to improve the stability of *Saccharomycopsis fibuligera* α-amylase [200], enabling a 5 °C increase in the enzyme’s optimal temperature. Srivastava [201] and Klibanov [202] propose that polysaccharides attached to enzymes during conjugation provide rigidity and hydration, thereby improving their stability. In this regard, Villalonga et al. [203] conjugated porcine pancreatic α-amylase (EC 3.2.1.1) with carboxymethylcellulose. As a result, the specific activity of the conjugate decreased by 54% compared to the native enzyme. However, the thermostability of the conjugated α-amylase improved significantly. While the native enzyme became inactive at 55 °C, the conjugate exhibited a complete loss of activity from 70 °C onwards. According to Villalonga et al. [203], the conjugate showed greater resistance to denaturing agents such as urea and sodium dodecyl sulfate. It is worth noting that such improvements are not typically achieved using traditional protein engineering techniques. However, to date, no chemical modification-based strategy has been employed in the thermostabilization of SIase.

## Figures and Tables

**Table 1:** Information on reported SIase crystal structures.

Microbial Source	Sucrose Isomerase	Mutation	PDB ID	Interacted Chemical	Resolution (Å)	References
*Pseudomonas mesoacidophila* MX-45 (*Rhizobium* sp. MX-45)	MutB	Non-mutant	2PWH	native	2.0	[56]
		Non-mutant	1ZJA	MutB-Tris	1.6	
		Non-mutant	2PWD	MutB-deoxynojirimycin	1.8	
		Non-mutant	2PWG	MutB-castanospermine	2.2	
		D200A	2PWF	MutB-glucose	1.8	
		E254Q	2PWE	MutB-sucrose	2.0	
		Non-mutant	1ZJB	MutB-Tris	1.8	[57]
		A258V	4GO8	MutB-Tris	2.15	
		D415N	4GO9	MutB-Tris	2.2	
		D200A-D415N (inactive enzyme)	4HA1	MutB-isomaltulose-glucose-Ca^2+^	2.2	[58]
		Non-mutant	4H8V	MutB-trehalulose-Ca^2+^	1.95	
		D200A-D415N (inactive enzyme)	4H8U	MutB-trehalulose-glycerol-Ca^2+^	2.0	
		E254Q-D415N (inactive enzyme)	4H8H	MutB-SO_4_^2−^-glycerol-Ca^2+^	2.0	
		D200A-D415N (inactive enzyme)	4H7V	MutB-glycerol-glucose-Ca^2+^	1.8	
		R284C	4H2C	MutB-glycerol-Ca^2+^	1.7	
		R284C	4GIN	MutB-glycerol-Ca^2+^	1.9	
		F164L	4GIA	MutB-Tris-glycerol-Ca^2+^	2.01	
		F164L	4GI9	MutB-Tris-glycerol-Ca^2+^	2.15	
		F164L	4GI8	MutB-Tris-glycerol-Ca^2+^	1.95	
		F164L	4GI6	MutB-Tris-glycerol-glucose-Ca^2+^	2.15	
*Erwinia rhapontici* NX-5	NX-5	Non-mutant	4HOW	(NX-5)-glycerol-Ca^2+^	1.7	[59]
		Non-mutant	4HOX	(NX-5)-Tris-glycerol-Ca^2+^	2.0	
		D241A	4HOZ	(NX-5)-glucose-glycerol-Ca^2+^	2.0	
		E295A	4HP5	(NX-5)-glucose-glycerol-Ca^2+^	2.0	
		E295Q	4HPH	(NX-5)-sucrose-glycerol-Ca^2+^	1.7	
*Protaminobacter rubrum* CBS574.77	SmuA	Non-mutant	3GBD	SmuA-C_6_H_5_O_7_^3−^-ethylene glycol	1.95	[60]
		Non-mutant	3GBE	SmuA-C_6_H_5_O_7_^3−^-ethylene glycol-deoxynojirimycin	1.7	
*Klebsiella* sp. LX3	PalI	Non-mutant	1M53	No information	2.2	[61]

**Table 2:** SIase with different general characteristics.

Isoform	T_op_ (°C)	pH	Specific Activity (U/mg)	Km (mM)	k_cat_/Km (mM^−1^·s^−1^)	Isomaltulose Ratio (%)	References
Wild-type PalI NX-5	30	6	423	222	NR	83	[87]
Recombinant PalI NX-5	30	5	NR	257	NR	87	[39]
Recombinant PalI NX-5	30	6	483.8	255.1	2.2	78	[54]
Wild-type PalI D12	40	6	19.8	138	NR	65.7	[40]
Wild-type NCPPB 1578	30	NR	4.11 ^a^	280	NR	85	[88]
Recombinant CBS 574.77	35	NR	NR	32.4	1301	88.5	[86]
Recombinant PalI NK33	35	6	2362	42.7	NR	76.8	[49]
Recombinant UQ68J	35	5	562	39.9	17.9	91	[51]
Recombinant UQ14S	35	6	351	76	6.2	66	[51]
Recombinant PalI LX3	35	6	328	54.6	0.27	83	[85]
Wild-type ATCC15928	30	6.2	120	65	NR	72.6	[41]
Recombinant AS9	30	6	957.5	30.1	33	76.3	[55]
Recombinant Ejp617	40	6	118.87	69.28	NR	80.5	[89]
Recombinant FMB-1	50	5–6	49	NR	NR	78	[50]
Recombinant Pal-2	40	5.5	286.4	62.9	NR	81.7	[48]

^a^ Activity refers to total proteins, using osmotic shock as an extraction method. NR—Not reported.

**Table 3:** Characteristics of mutant sucrose isomerases with improved thermostability.

Isoform	Modification	Strategy for Thermostabilization	Stabilizing Interaction	T_op_ (°C)	Half-Life (min)	Specific Activity (U/mg)	Km (mM)	k_cat_/Km (mM^−1^·s^−1^)	References
PalI NX-5	Glycosylation	B-factor analysis and glycosylation site engineering.	Strengthening of the hydrogen-bonding network and glycosylation of the flexible terminal C region.	30	10.1 ^a^	483.8	255.1	2.21	[54]
PalI NX-5	Glycosylation—K174Q			35	22.3 ^a^	529.9	241.9	2.55	
PalI NX-5	Glycosylation—L202E			35	17.5 ^a^	509.1	234.9	2.52	
PalI NX-5	Glycosylation—K174Q/L202E			35	29.2 ^a^	509.3	231.2	2.57	
PalI AS9	Native	B-factor analysis	Strengthening of the hydrogen bridge network	30	39.2 ^b^	957.5	30.1	33	[55]
PalI AS9	E175N			35	90.2 ^b^	1017.6	28.1	45.6	
PalI AS9	K576D			35	69.8 ^b^	1045.7	29.5	34.4	
PalI AS9	E175N/K576D			35	300 ^b^	1218.9	26.8	39.4	
PalI LX3	Native	Proline theory	Loop stabilization	35	1.81 ^c^	328	54.6	0.27	[85]
PalI LX3	E498P			40	9.45 ^c^	350	NR	0.29	
PalI LX3	E498P/R310P			40	13.61 ^c^	340	NR	0.31	

^a^ The enzymes were incubated in 50 mM citric acid/sodium phosphate buffer (pH 6.0) at 40 °C. ^b^ The enzymes were incubated in 50 mM citric acid/sodium phosphate buffer (pH 6.0) at 45 °C. ^c^ The enzymes were incubated in 0.1 M citrate-phosphate buffer (pH 6.0) at 50 °C.

## Data Availability

Not applicable.
